# Preparation of MSZ Hydrogel and Its Treatment of Colitis

**DOI:** 10.3389/fphar.2021.706401

**Published:** 2021-10-08

**Authors:** Yanhong Wu, Shangyong Li, Mengfei Jin, Dandan Li, Zihan Zhou, Haiqing Hou, Yantao Han

**Affiliations:** ^1^ School of Basic Medicine, Qingdao University, Qingdao, China; ^2^ Department of Clinical Laboratory, Qilu Hospital(Qingdao), Cheeloo College of Medicine, Shandong University, Qingdao, China

**Keywords:** MSZ, HP-β-CD, ALG, CS, κ-Car, hydrogel, colitis

## Abstract

In order to control the release of mesalazine (MSZ) in the gastrointestinal tract to achieve better pharmacological effects in the colon, in this study, MSZ was added to hydroxypropyl-β-cyclodextrin (HP-β-CD) to form a water-soluble HP-β-CD/MSZ inclusion complex. Then, the inclusion compound was loaded into the structure of the bilayer polyelectrolyte complex microsphere formed by alginate (Alg), chitosan (Cs), and kappa carrageenan (κ-Car) as the hydrogel carrier, and the hydrogel beads with colon-specific release MSZ after oral administration were formed. The formed hydrogel beads have different swelling capabilities in different pH media and have the greatest swelling degree under pH 7.4. The encapsulation efficiency and drug loading of hydrogel beads can reach up to 83.23 and 18.31%, respectively, and the size of hydrogel beads can be reduced to less than 1 mm after drying, so that the size of oral administration can be reached. *In vivo* experiments also showed that the formed hydrogel beads had a better therapeutic effect on colitis than free drugs, and the microspheres were biodegradable, so the double-layer pH-sensitive microspheres could be effectively used in colon-targeting drug delivery.

## Introduction

Ulcerative colitis (UC) is an incurable inflammatory disease of the colon with a rapid increase in incidence worldwide, and the risk of UC patients developing colon cancer has also increased significantly (Si et al., 2016). Therefore, the prevention and treatment of UC has important clinical significance in reducing the sufferings of patients, improving the quality of life of patients, and reducing the occurrence of colon cancer (Naeem et al., 2020). At present, drug therapy is still the main method in the treatment of UC. Among the therapeutic drugs, mesalazine (MSZ) is currently used as the first-line therapy for UC (Duan et al., 2016). MSZ is an anti-inflammatory drug, which is usually used in the treatment of mild to moderate active ulcerative colitis. By inhibiting the synthesis of prostaglandins that cause inflammation and the formation of inflammatory mediators, leukotrienes, it has a significant inhibition effect on intestinal mucosal inflammation (Tang et al., 2018). MSZ has been proven to be a potential scavenger of reactive oxygen species, which plays an important role in the pathogenesis of inflammatory bowel disease, neutrophil functional damage, cyclooxygenase, and lipoxygenase (Mladenovska et al., 2007). However, the therapeutic effect of MSZ is greatly limited due to its poor water solubility and a low dissolution rate, and MSZ is absorbed through the mucosa of the upper gastrointestinal tract (stomach and small intestine), which impairs its pharmacological effects in the colon and causes side effects, including nephrotic syndrome and hepatitis (Zou et al., 2005; Chen et al., 2012; Hu et al., 2012). Therefore, it is particularly important to develop a new type of MSZ drug delivery system to maximize its therapeutic effect. Cyclodextrin is widely used in medicine, food, cosmetics, and other fields because of its hydrophobic inner cavity and hydrophilic outer cavity (Varan et al., 2017). In the research of pharmaceutical preparations, cyclodextrin and its derivatives can effectively include small drug molecules to improve the solubility, dissolution rate, stability, and spectroscopic properties of the drug (Yadav et al., 2009; Elbashir et al., 2015). For example, HP-β-CD is a hydroxylated derivative of β-CD, with good biocompatibility, almost no hemolysis and nephrotoxicity, and due to its surface activity and cavity depth it can effectively improve the stability and solubility of MSZ (Tang et al., 2018).

Oral administration is the most common way in our daily life compared with other ways of administration, such as injection and perfusion; however, after oral administration of ordinary pharmaceutical preparations, the drugs are generally released and absorbed in the stomach and small intestine and the amount of drugs reaching the colon is relatively small, so the effective drug concentration required for treatment is often not reached at the lesion site (Niu et al., 2019). Colon-specific drug delivery systems (CDDSs) have received extensive attention from researchers because they can target and release drugs in the colon and have a good therapeutic effect on colon diseases (Philip and Philip, 2010; Amidon et al., 2015). The pH-sensitive oral CDDS is currently the most widely used CDDS, which is mainly designed based on the difference in pH of the gastrointestinal tract. In general, the pH of the stomach is close to 1.2, the pH of the duodenum is about 4.5, the pH of the small intestine is about 6.0–6.8, the pH of the end of the small intestine is 6.8–7.0, and the pH of the colon is 6.5–7.5. Hydrogels are considered a suitable oral drug delivery system because they have many unparalleled advantages, including high drug loading efficiency, better stability, and simple preparation (Pasparakis and Bouropoulos, 2006; Niu et al., 2019). Through hydrogels of different designs, colon-specific drug release can be achieved. That is to say, in the upper part of the gastrointestinal tract (low pH environment), the carrier material does not dissolve and swells to a small degree, resulting in less drug release, while in the colon (high pH environment), the carrier material gradually dissolves and the drug release increases (Lin et al., 2005; Omer et al., 2016; Duan et al., 2017).

Marine biopolymers are a kind of multipurpose natural polymers. Due to their low toxicity, biocompatibility, biodegradability, low cost, renewability, and richness, they are being widely developed and applied in the fields of biomedicine and pharmaceuticals (Dey et al., 2013; Amiri et al., 2017). One of them, chitosan (Cs), is a cationic polymer extracted from chitin, consisting of glucosamine and N-acteyl glucosamine units through a β-(1→4) bond group (Amiri et al., 2021). Sodium alginate (Alg) is composed of α-1,4-L-guluronic acid (G) sugar units and β-1,4-D-mannuronic acid (M) sugar units alternately. It is a linear, anionic polysaccharide (Wang et al., 2019; Zhou et al., 2018). Similar to Alg, Car is a kind of sulfated anionic marine biopolymer, in which kappa carrageenan (κ-Car) has excellent gelling properties as well as potential application in the medical field due to its antioxidant, antitumor, and antibacterial activities (Guzman-Villanueva et al., 2013; Luo and Wang, 2014). The interaction between the positively charged amine group of Cs and the negatively charged Alg and κ-Car can form a polyelectrolyte complex (PEC) sensitive to pH (Grenha et al., 2010; Sun et al., 2019).

In this study, MSZ was added to annular HP-β-CD to form a water-soluble CD-MSZ inclusion compound, which was encapsulated in a hydrogel composed of Alg, Cs, and κ-Car. The drug release test showed that the formed hydrogel has a sustained release effect under GIT conditions. The CCK-8 test confirmed that the hydrogel is basically nontoxic to Raw 264.7 macrophages. The results of *in vivo* experiments showed that oral administration of the hydrogel had a better therapeutic effect on DSS-induced colitis rats. The hydrogel is expected to be a potential oral drug delivery system for MSZ to treat UC (Javanbakht et al., 2020).

## Materials and Methods

### Materials

Chitosan (Cs), sodium alginate (Alg), and kappa carrageenan (κ-Car) were obtained from Sigma-Aldrich (St. Louis, MO, United States). Dextran sodium sulfate (DSS) was supplied from MP Biomedicals (Irvine, CA, United States). MSZ, β-CD, and HP-β-CD were purchased from Solarbio (Beijing, China). Male C57BL/6 mice (8 week old, 18–22 g) were provided by Pengyue Laboratory Animal Technology Co., Ltd. (Jinan, China). Myeloperoxidase (MPO) kits were obtained from Nanjing Jiancheng Bioengineering Institute (Nanjing, China). The Cell Counting Kit-8 (CCK-8) was obtained from Dongren Chemical Technology Co., Ltd. (Shanghai, China). The hematoxylin and eosin (H&E) staining kit was purchased from Solarbio (Beijing, China). The total antioxidant capacity assay kit with the ABTS method was obtained from Beyotime Biotechnology. Rhodamine B isothiocyanate (RBITC) was purchased from Macklin (Shanghai, China).

### Increase and Comparison of Solubility of MSZ by β-CD and HP-β-CD

The formation of a β-CD/MSZ inclusion complex: the β-CD/MSZ inclusion complex was prepared by the co-precipitation method. β-CD was dissolved in deionized water, continuously stirred, and heated to 60°C to completely dissolve β-CD, as the water phase; MSZ was dissolved in ethanol by ultrasound (250 W, 40 KHz), as an organic phase; added the organic phase dropwise to the water phase at a speed of 1 ml/min, ultrasonic mixing (250 W, 40 kHz) for 30 min, and stirred at room temperature for 24 h; and the prepared suspension solution was centrifuged to remove unincorporated free MSZ, and then dried obtain β-CD/MZS inclusion compound powder.

The formation of HP-β-CD/MSZ inclusion compound: in short, at room temperature, under continuous stirring (magnetic stirrer stirring), added MSZ powder to 2:1 (v/v) acetone/water solution until completely dissolved. The HP-β-CD was completely dissolved in water, and then the completely dissolved MSZ was added dropwise to the HP-β-CD aqueous solution. The mixed solution was first continuously stirred at 40°C for 5 h with a magnetic stirrer, and then the temperature was raised to 60°C to remove the organic solvent in the solution. The final solution was filtered to remove the unincorporated MSZ, the filtered solution was cooled to room temperature, and then it was frozen at −20°C for a certain period of time and then transferred to −80°C. Finally, the completely frozen solution was dried in a freeze dryer to obtain HP-β-CD/MSZ inclusion compound powder (Tang et al., 2018).

The dried β-CD/MSZ inclusion compound and HP-β-CD/MSZ inclusion compound powder were respectively dissolved in distilled water for solubility measurement and comparison.

### Fabrication of HP-β-CD/MSZ/Alg/Cs/κ-Car Hydrogel Beads

All beads were manufactured according to the description in the literature, with some modifications (Pasparakis and Bouropoulos, 2006; Sun et al., 2019). First, HP-β-CD/MSZ/Alg microbeads were formed by ion gelation. Then, dissolved an appropriate amount of HP-β-CD/MSZ inclusion compound powder in distilled water, then added a certain proportion of Alg, and stirred to make it fully dissolved. Calcium chloride was dissolved in distilled water, and then used a peristaltic pump to drop the solution containing HP-β-CD/MSZ and Alg into the calcium chloride solution to ensure the distance between the surface of the calcium chloride solution and the outflow of the solution from the peristaltic pump was 8 cm. In this process, the HP-β-CD/MSZ inclusion compound was encapsulated in the matrix of calcium alginate hydrogels. The microbeads formed by adding dropwise to the calcium chloride solution were slowly stirred and kept in the calcium chloride aqueous solution, and the microbeads were solidified at room temperature for 20 min (Zhang et al., 2012). We removed the beads and washed with distilled water to remove calcium chloride from the surface of the beads. The process was repeated three to four times. Then, an appropriate amount of chitosan was dissolved in the acetic acid solution to prepare the chitosan solution. Then transferred the newly obtained HP-β-CD/MSZ/Alg microbeads to the chitosan solution and kept it under gentle magnetic stirring for 20 min to achieve the purpose of CS coating HP-β-CD/MSZ/Alg microspheres. Then the beads were separated from the solution and gently rinsed with distilled water to remove excess unreacted Cs. Finally, because of the ion interaction between -SO_3_^−^ in κ-Car and -NH_3_^+^ in Cs, the HP-β-CD/MSZ/Alg/Cs beads with the outermost layer of Cs can be coated again by κ-Car. After the κ-Car was completely dissolved in distilled water, we added HP-β-CD/MSZ/Alg/Cs beads to the κ-Car solution and stirred gently with a magnetic stirrer at 40°C for 30 min. We kept HP-β-CD/MSZ/Alg/Cs/Car beads in KCl solution for 10 min. After removing the water condensed beads, we rinsed it with distilled water. The obtained hydrogel beads were dried at room temperature for later use (Chen et al., 2012). The production of blank hydrogels was that the HP-β-CD/MSZ inclusion compound was not dissolved in distilled water at the beginning, while Alg was dissolved directly in distilled water. Then it was added into calcium chloride solution, the following production was the same as the above process.

### Characterization of the HP-β-CD/MSZ Inclusion Complex and Hydrogel Beads

The structure and morphology of the HP-β-CD/MSZ inclusion complex and hydrogel beads were characterized using a particle size analyzer, a high resolution transmission electron microscope (TEM), and a scanning electron microscope (SEM).

### Determination of Drug Encapsulation Efficiency (EE) and Loading Content (LC)

In order to determine the encapsulation efficiency and drug loading of hydrogel microbeads, accurately weighed dry HP-β-CD/MSZ/Alg/Cs/Car microbeads were placed in a phosphate buffer solution of pH 7.4. Then the swelling was carried out under the conditions of 30°C and 150 rpm shaking table. After 15 h, the swollen beads were pulverized by ultrasonic wave, and then placed on the shaking table to continue shaking. After 24 h, the suspension was filtered. The amount of MSZ (298 nm) in the filtrate was measured by an ultraviolet-visible spectrometer. All samples were analyzed in triplicate. The encapsulation rate (%) and drug loading (%) of MSZ in microbeads were calculated according to the following equations (Maiti et al., 2010).

EE(%)=(weight of MSZ in beads/theoretical MSZ loading)×100

LC(%)=(weight of MSZ in beads/weight of beads)×100

### Fourier Transforms Infrared (FTIR) and X-Ray Diffraction Analysis

The dried unloaded and drug-loaded hydrogel balls were crushed in a mortar with a pestle. The crushed materials, pure Alg, Cs, κ-Car, HP-β-CD, MSZ, physical mixture of HP-β-CD and MSZ, and the HP-β-CD/MSZ inclusion complex were mixed with KBr (infrared spectrum grade), respectively, dried and pressed into a shape for Fourier transform infrared spectroscopy. Fourier transform infrared spectrometer was used to record infrared spectrum (spectral scanning is performed in the wavelength range of 400–4000 cm^−1^).

X-ray diffraction (XRD) spectra of MSZ, HP-β-CD, the physical mixture of HP-β-CD and MSZ, the HP-β-CD/MSZ inclusion complex, and several substances composing hydrogels were determined by XRD (Bruker D8 Advance). XRD data were collected at a 40 kV tube voltage and 40 mA tube current. The scanning regions of diffraction angle 2θ were 5°–80° at a scan rate of 5°/min.

### *In Vitro* Drug Release

According to the physiological characteristics of human GIT (the pH of the stomach, duodenum, small intestine, and colon are 1.2, 4.5, 6.8, and 7.4, respectively), in order to measure the *in vitro* release of MSZ, a suitable amount of dry hydrogel beads containing the HP-β-CD/MSZ complex were immersed in 100 ml of pH 1.2, 4.5, 6.8, and 7.4 medium at 37°C, respectively, and gently shaken (75 rpm). At a predetermined time point, 1 ml of release medium was taken out and replaced with fresh buffer solution. The extracted solution was filtered by 0.22 μM filter, the absorbance value was measured at 298 nm by an UV spectrophotometer, and calculated the cumulative release of MSZ. For simulating drug release *in vivo*, the hydrogel beads were first released in pH 1.2 HCl buffer for the first hour, then released in pH 4.5 PBS for 2 h, in pH 6.8 PBS for 2 h, and finally in pH 7.4 PBS until the end of the experiment. For the release of the MSZ and the HP-β-CD/MSZ inclusion complex, an appropriate amount of the MSZ and the HP-β-CD/MSZ inclusion complex were dispersed in the solution medium and placed in a dialysis tube, respectively, and then the dialysis tube was placed in 100 ml solution medium at 37°C and stirred continuously. Samples were taken at the same time points. The *in vitro* release study was repeated three times (Duan et al., 2016; Duan et al., 2017; Ceylan et al., 2020).

### Swelling Ratio of Dried Hydrogel Beads

For the swelling behavior of microbeads, four different pH buffer media (pH 1.2, 4.5, 6.8 and 7.4) were used to study. The accurately weighed microbeads were immersed in a buffer medium and expanded at 37°C. The beads were separated from the swelling medium at a specific time, dried with filter paper to remove excess water from the surface, and then weighed immediately on an electronic balance. Each experiment was repeated three times (Lin et al., 2005). The swelling degree of the sample is determined according to the following formula:

$$\text{Swelling}\;\text{degree}\left(\text{\%}\right)=\left[\left({\text{M}}_{\text{t}}-{\text{M}}_{\text{0}}\right)/{\text{M}}_{\text{0}}\right]\text{×}100,$$

where M_t_ is the weight of the swollen microbeads and M_0_ is the initial dry weight of the microbeads.

### Cytotoxicity Assays

In this experiment, Raw 264.7 macrophages, HT-29 cells, and a Cell Counting Kit-8 (CCK-8) were used to study the cell proliferation–toxicity detection experiment of HP-β-CD/MSZ inclusion compound and hydrogel spheres (Niu et al., 2019; Pan et al., 2019). The cells were cultured in a culture medium containing 10% (v/v) serum, 1% antibiotics, and 89% medium, and cultured in a humidified environment with 5% CO_2_ at 37°C. For blank hydrogels and drug-loaded hydrogels, first immersed 20 mg of hydrogel spheres in 10 ml of PBS (pH 7.4) in advance, and swelled for 24 h. In addition, the hydrogel spheres were broken by an ultrasonic cell disruptor. The resulting mixture was diluted 10-fold with cell culture medium. The cells were seeded in a 96-well tissue plate at a density of approximately 3 × 1000 per well and different concentrations of the HP-β-CD/MSZ inclusion complex (200, 100, 50, and 25 mg/ml), a blank hydrogel, and a drug-loaded hydrogel were added, respectively, and then incubated with the cells for 24 h and added CCK-8; after 2 h, we recorded the optical density (OD) at 450 nm. Experiment was carried out in triplicate.

### Animal Maintenance

Male C57BL/6 mice (8 week old, 18–22 g) came from an animal supplier approved by Qingdao University and were provided by Pengyue Laboratory Animal Technology Co., Ltd. (Jinan, China). The indoor temperature and relative humidity for feeding mice were controlled at 24–26°C and 60–80%, respectively, and the light/dark cycle was maintained for 12 h in the room. In the early stage, these animals allowed unlimited access to standard mouse food and distilled water. All mice were acclimatized to the facility for 1 wk, and then randomly divided into six groups (six in each group) (Han et al., 2019).

### Mice UC Model and Drug Treatment

In order to establish the C57BL/6 mouse colitis model, except for the first group of mice which were given normal water, the mice in the other groups were given 2.5% DSS freely, and all mice were free to eat. The corresponding drugs were used for treatment on the fourth day after DSS administration. Specifically, the first group was the normal group (continued to give distilled water), the second group was the inflammation group (DSS group), the third group was the commercially available mesalazine group (positive drug), the fourth group was the drug-loaded hydrogel group (gavage), the fifth group was the HP-β-CD/MSZ inclusion complex direct gavage group, and the sixth group was the blank hydrogel group. After a total of 7 days of DSS administration, the distilled water containing DSS was replaced by pure distilled water. The flow chart is shown in Supplementary Figure S1. The normal mice and DSS mice were given the same intragastric stimulation every day. The mice were anesthetized to death 24 h after the last drug treatment, and on the 10th day, the spleen was isolated and the weight gain was recorded as an index of inflammation. The tissues from cecum to anus and eyeball blood were collected, and before segmenting the colon for histological analysis, pictures were taken to measure the length of the colon and rectum. Colonic tissue samples (1 cm) were excised and flushed with buffer to remove colonic contents and then placed in 4% paraformaldehyde solution for histopathological analysis (Han et al., 2019; Naeem et al., 2018; Zhou XL. et al., 2020).

### Evaluation of Colitis Severity by Body Weight Changes and the Disease Activity Index (DAI)

On each day of the experiment, the mice were examined and weighed to see if there were any signs of disease, and the pathological process of acute colitis was evaluated by the disease activity index (DAI) (Zhou XL. et al., 2020). Three parameters were used to monitor the clinical course of colitis daily: weight loss, stool consistency, and anal bleeding. In short, the inflammation score was 0–4, weight loss was 0 points, 1%–5% weight loss was one point, 5–10% weight loss was two points, 10–20% weight loss was three points, and >20% weight loss was four points. For the consistency of stool, a score of 0 was given to well-formed pellets, two points were given to paste-like and semi-formed stools that did not stick to the anus, and four points were given to liquid stools that stick to the anus. Bleeding was divided into 0 points for no blood, two points for positive findings, and four points for heavy bleeding. The average of these scores constitutes the clinical score, ranging from 0 (healthy) to 4 (maximum colitis) (Naeem et al., 2018).

### Antioxidant Activity

MSZ is known as an anti-inflammatory drug and has strong antioxidant capacity. The total antioxidant capacity test kit (2,20-azino-bis(3-ethylbenzothiazoline-6-sulfonate) (ABTS method) was used for different concentrations of HP-β-CD/MSZ inclusion compound (100, 50, 25, 5 mg/ml) and the serum of the eyeball blood of the mouse experimental group were tested for their antioxidant capacity. ABTS is oxidized to green ABTS^+^ under the action of appropriate oxidants. The production of ABTS^+^ will be inhibited in the presence of antioxidants. By measuring the absorbance of ABTS^+^ at 734 nm, the total antioxidant capacity of the sample can be determined and calculated.

### Myeloperoxidase (MPO) Activity Assay

The severity of colitis can be quantified by measuring myeloperoxidase activity, which is a reliable indicator of inflammation caused by the infiltration of activated neutrophils into inflamed tissues (Mladenovska et al., 2007; Zhou X. et al., 2020). After the colon tissue was minced and homogenized, the MPO activity was measured. Finally, the absorbance of the supernatant was measured at 450 nm by spectrophotometry. H_2_O_2_ was decomposed into 1 unit of enzyme activity per gram of wet piece of colon tissue in the reaction system at 37°C. The results were expressed in U/g tissue.

### Histopathologic Analysis

For histopathological analysis, a small part of the colon from mice in the healthy control group, the DSS control group, and the drug treatment group was first fixed in formalin, and finally embedded in paraffin. The slices (5 μm thick) were cut with a microtome, stained with hematoxylin and eosin (H&E), and then analyzed by a light microscope (Zhou et al., 2020b).

### *Ex vivo* Imaging

A small animal *in vivo* imaging system was used to track the colon-specific drug delivery potential of the hydrogel after oral administration. The near-infrared fluorescent dye RBITC was loaded into the hydrogel as a fluorescent probe. After fasting for 6 h, mice with colitis were given the same dose of the RBITC-labeled hydrogel and hydrogel without RBITC-labeled. After 6 and 12 h of oral administration, a small animal *in vivo* imaging system was used to perform *in vivo* imaging of the mice at an excitation wavelength of 558 nm and an emission wavelength of 586 nm, and the cecum to anus tissues were taken for imaging after the death of the anesthesia administration (Yuan et al., 2015).

### Statistical Analysis

For repeated experiments, the experiment needs to be repeated at least three times to ensure the accuracy of the data, so the repeated experiments are made in triplicate; the experimental data obtained is expressed as the mean ± standard deviation (SD), and the statistical significance is defined as *p* < 0.05. Statistical analysis was performed using GraphPad Prism 5.0 for Windows (GraphPad Software, La Jolla, CA, United States). All data are presented as the mean ± SD. The statistical significance between multiple groups was calculated by one-way analysis of variance (ANOVA). The sample size of mice is calculated using one-way analysis of variance. We used PASS 15.0 software to calculate the sample size of 36 mice, six in each experimental group (six experimental groups), and the power was above 0.9. So it is enough to say that there are six mice in each group.

## Results and Discussion

### Comparison of β-CD and HP-β-CD for Increasing the Solubility of MSZ

Due to its hydrophobic inner cavity and hydrophilic outer cavity, β-CD can effectively enclose small drug molecules to improve the solubility, the dissolution rate, and stability of the drug (Shelley and Babu, 2018), so it can effectively improve the stability and solubility of MSZ. The solubilization effect of β-CD and HP-β-CD on MSZ was compared by a phase dissolution method, and it was found that the solubilization effect of HP-β-CD on MSZ was significantly better than β-CD. HP-β-CD has better water solubility than β-CD, good biocompatibility, and almost no hemolysis and nephrotoxicity; therefore, HP-β-CD was selected as the inclusion material of MSZ.

### Characterization

#### Characterization of the HP-β-CD/MSZ Inclusion Complex

**FIGURE 1:**
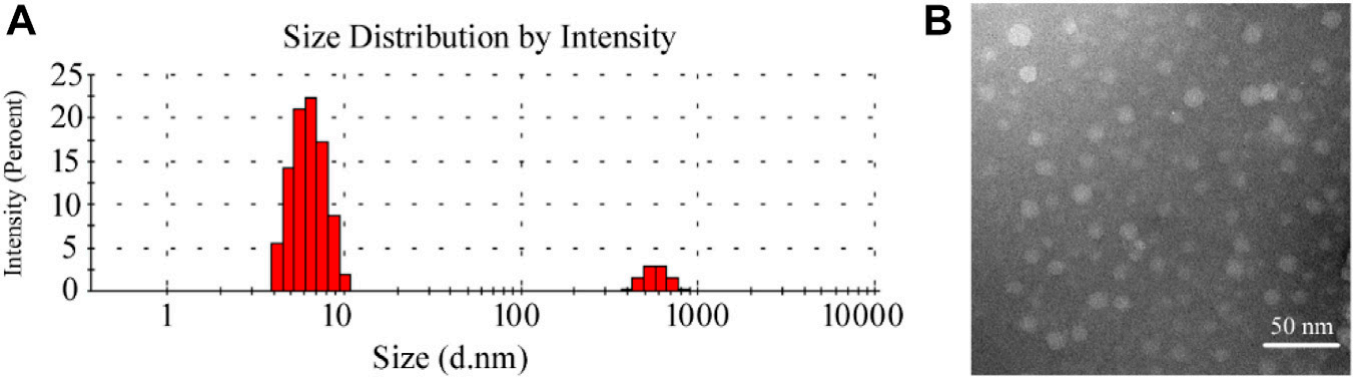
Characterization of the HP-β-CD/MSZ inclusion complex. **(A**,**B)** are the particle size distribution and TEM diagram, respectively.

Figure 1 shows the particle size distribution and TEM diagram of HP-β-CD/MSZ inclusion compound, respectively. HP-β-CD has a lipophilic central cavity, and MSZ is absorbed into the cavity by forming a water-soluble inclusion compound. It can be seen from the figure that the average particle size after HP-β-CD clathrates MSZ is about 10 nm. The aqueous solution of HP-β-CD/MSZ inclusion compound is easy to self-assemble to form nanoscale aggregates. It can be seen by TEM that some of these were small spherical particles, and some seem to have slightly irregular shapes.

#### Morphological Analysis of Beads

**FIGURE 2:**
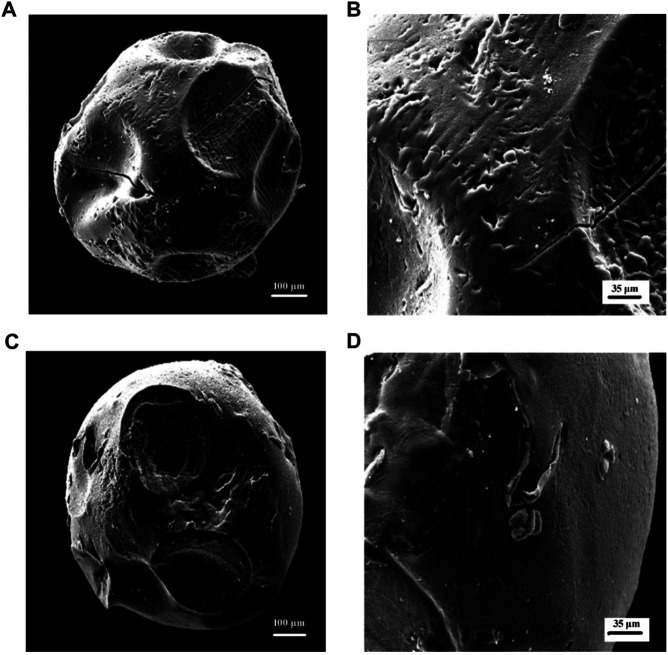
**(A**,**B)** are SEM micrographs (300 x) (100k x) of Alg hydrogel beads in dry condition; **(C**,**D)** are SEM micrographs (300 x) (100k x) of Alg/Cs/Car hydrogel beads in dry condition.

The surface structure of the hydrogel beads may affect the migration of water into the gel beads and the release of drugs. Therefore, the surface morphology of the hydrogel beads was analyzed by SEM (Gholamali and Yadollahi, 2020). Generally, hydrogel wet beads were spherical, about 1.9–2.2 mm in diameter, and had a smooth surface. Figures 2A–D respectively show the scanning electron micrographs of the dried pure calcium alginate hydrogel and the hydrogel beads formed after Cs and κ-Car are wrapped around them at 300x and 1.00x magnifications. Due to the partial collapse of the polymer network during the dehydration process, the surface morphology of the dried microbeads changed significantly. After drying, the diameter of the test beads was mostly reduced to less than 1 mm, and the spherical state was not completely maintained as, obviously, the spherical shape of the microspheres disappeared after drying. The dry pure calcium alginate hydrogel showed a rough surface, a collapsed center with small folds and many cavities (Figure 2B). It can be seen from Figure 2D that the hydrogel beads coated with Cs and κ-Car had a reduced roughness compared to pure Alg hydrogels, and the surface had become relatively smooth and compact surface, and there were fewer cavities. These changes may be due to the effect of Cs with Alg and κ-Car. These morphological characteristics are closely related to the water exchange and swelling of the hydrogel. After the hydrogel is destroyed in the proper part, the HP-β-CD/MSZ inclusion complex is released from the hydrogel and acts on the body to exert pharmacological effects. When hydrogels are used in the treatment of colitis, their swelling properties are essential for drug transfer.

#### FTIR Analysis

**FIGURE 3:**
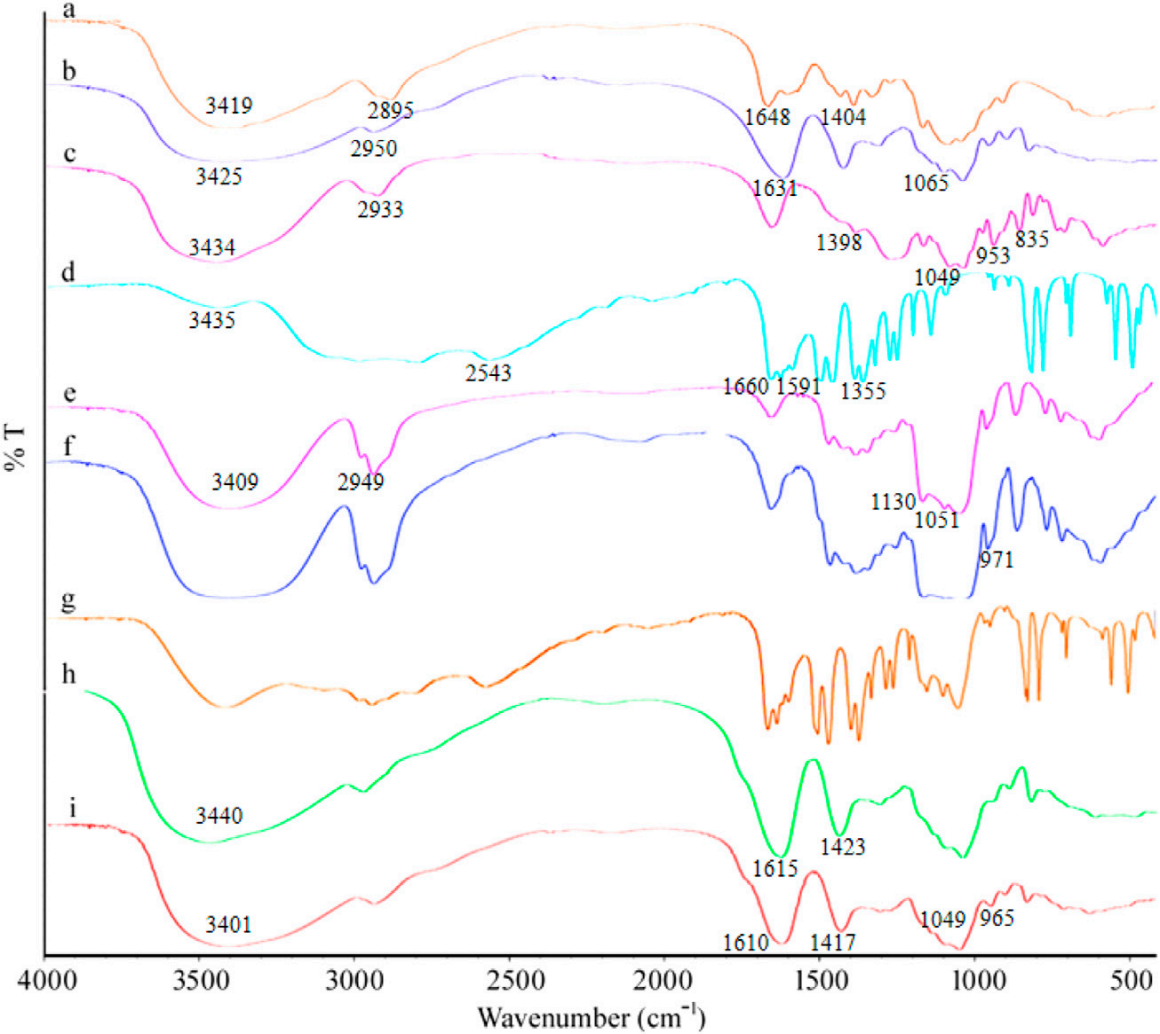
FT-IR spectra. a, Alg; b, Cs; c, κ-Car; d, MSZ; e, HP-β-CD; f, inclusion complex of MSZ and HP-β-CD; g, physical mixture of MSZ and HP-β-CD; h, blank hydrogel; and i, drug loaded hydrogel.

It can be seen from the FTIR spectra (Figure 3) of MSZ, HP-β-CD, their physical mixtures, and the HP-β-CD/MSZ inclusion complex that MSZ had characteristic peaks at 3435 cm^−1^ (-NH and -OH tensile vibration), 2543 cm^−1^ (-NH2 vibration), 1660 cm^−1^ (C=O stretching), 1591 cm^−1^ (C=C vibration), and 1355 cm^−1^ (C-N stretching). Due to the existence of O-H stretching vibration, the FTIR spectrum of HP-β-CD had an obvious absorption band at 3409 cm^−1^ and C-H stretching vibration at 2949 cm^−1^, and the symmetric and asymmetric stretching vibrations of C-O-C at 1130 and 1051 cm^−1^, respectively. When the inclusion compound was formed, some peaks disappear or their intensity was weakened, the wave number shifted and new bands appeared. For example, the obvious MSZ peak at 2543 cm^−1^ disappears, and the broad absorption peak of HP-β-CD appears in the FTIR spectrum of the inclusion compound. These changes indicated that MSZ was deeply inserted into the HP-β-CD cavity; however, the infrared spectrum of physical mixture was a simple superposition of the characteristic peaks of MSZ and HP-β-CD, which indicated that pure physical mixing will not cause the interaction between MSZ and HP-β-CD. From the Fourier transform infrared spectra of several substances forming hydrogels, it can be seen from the figure that Alg had a wide band at 3419 cm^−1^ due to the stretching vibration of the O-H group. There was an absorption band at 2895 cm^−1^, which corresponds to the stretching frequency of the C-H group. Alg produces absorption bands at 1648 and 1404 cm^−1^, which are the characteristics of asymmetric stretching and symmetric stretching of -COO. The Cs spectrum showed the broadband produced by -NH_2_ at 3425 cm^−1^; the stretching of -OH had an absorption band at 2950 cm^−1^. The characteristic peak of Cs appeared at 1631 cm^−1^, which corresponds to the amide bond. The stretching of the C-O bond was shown at 1065 cm^−1^. The characteristic peaks of κ-Car were shown at 3434, 2933, 1398, 1049 cm^−1^, and so on. Compared with the individual substances, the characteristic peaks of the finally formed hydrogel showed significant differences. Due to the strong interaction between Cs and Alg and κ-Car, some peaks may move, weaken, or disappear. For the hydrogel loaded with HP-β-CD/MSZ, the characteristic absorption of HP-β-CD/MSZ was marked by other stronger signals from the drug-loaded hydrogel, which indicated that HP-β-CD/MSZ was loaded into the polymer network of the hydrogel.

XRD is one of the methods to characterize the formation of inclusion complexes between HP-β-CD and guest molecules. The crystalline nature of the guest molecule will change as it forms an inclusion compound with HP-β-CD. As shown in Supplementary Figure S2A, the MSZ exhibited sharp peaks at 2θ of 7.6°, 15°, 16.2°, and 31°, indicating its crystalline form. It can be seen from the figure that the X-ray diffraction pattern of HP-β-CD had a broad diffraction peak of 10° and 19.8° at 2θ, confirming its amorphous structure. The diffraction pattern of the physical mixture of HP-β-CD and MSZ was a simple superposition of the two components. No characteristic diffraction peaks were observed in the diffraction pattern of the HP-β-CD/MSZ inclusion complex, indicating that the complex was amorphous. They proved that MSZ exists in the cavity structure of HP-β-CD. For several substances that form hydrogels; it can be seen from Supplementary Figure S2B that Cs, as a semicrystalline biopolymer, had broad diffraction peaks at 10.2° and 20.5°. The XRD spectra of Alg and κ-Car did not show obvious crystalline peaks, indicating their amorphous nature. In the process of forming the hydrogel, the semicrystalline structure of chitosan was interrupted, and the formation of hydrogen bonds was not allowed. The amorphous structure of the hydrogel means that the ionic interaction between Cs with Alg and κ-Car leads to their excellent compatibility and promotes the interaction of Alg and κ-Car with Cs molecules.

#### Determination of Drug Encapsulation Efficiency (EE) and Loading Content (LC)

The EE (%) and LC (%) of the HP-β-CD/MSZ inclusion compound successfully loaded into Alg microbeads could reach 88.91 and 24.02%, respectively, while loading into the Alg/Cs/k-Car hydrogel, EE (%) and LC (%) were 83.23 and 18.31%, respectively. The decrease in EE and LC may be due to the increase in contact and washing time when Cs and k-Car were added, which caused part of the drug to be lost.

#### *In vitro* Drug Release Studies

**FIGURE 4:**
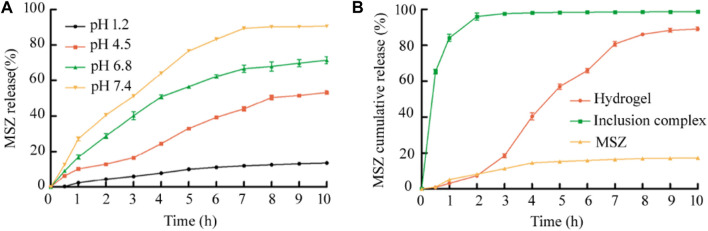
**(A)**The release rate of MSZ from the Alg/Cs/Car hydrogel under different pH medium conditions was compared; **(B)** *In vitro* cumulative release of the drug-loaded hydrogel, the HP-β-CD/MSZ inclusion complex, and free MSZ under simulated *in vivo* conditions. Data are mean ± SD (*n* = 3) of three independent experiments.

For the study of the drug release rate, the simulated gastric juice and three simulated intestinal juices were selected as the media to simulate the human GIT environment at 37°C, and the drug release under several media was studied first. As shown in Figure 4A, it can be seen from the experimental results that the pH value of the release medium has a great influence on the drug release performance. Among the four buffer solutions, it was obvious that the drug release rate is the lowest at pH 1.2, and the hydrogel beads release less than 14% within 10 h. With the increase of the pH of the medium, the cumulative release of MSZ increased. At pH 4.5 and pH 6.8, the cumulative release of MSZ was about 53 and 71%, respectively, and it could reach 90% at pH 7.4.

The next step was to conduct research under pH gradual conditions that simulate different parts of the real GIT. The results were the same as expected, and as shown in Figure 4B, free MSZ was slightly released from the dialysis tube and showed a cumulative release rate of less than 20% after a few hours, probably due to the poor solubility of MSZ in the dissolution medium. For the inclusion complex, MSZ showed the characteristics of rapid release at the beginning. Compared with the free MSZ and the HP-β-CD/MSZ inclusion complex, MSZ hydrogels showed significantly improved release characteristics. As shown in the figure, MSZ did not release in the first hour, but rarely in the next 2 h. In 2 h at pH 6.8, the release increased, and within a few hours at pH 7.4, the release basically reached a maximum. So it can be seen that MSZ in the hydrogel exhibited a pH-sensitive release, and the cumulative release increased with the increase of the pH value. When the pH is lower than 7.0, the release of the drug can be prevented. When the pH value changes to 7.4, which corresponds to the pH value of the ileocecal area and the colon, the remaining drugs can be released. Therefore, pH dependence can prevent the early release of the drug at the proximal end of GIT and avoid the complete release of the drug before it reaches the colon.

#### Swelling and pH-Sensitivity

**FIGURE 5:**
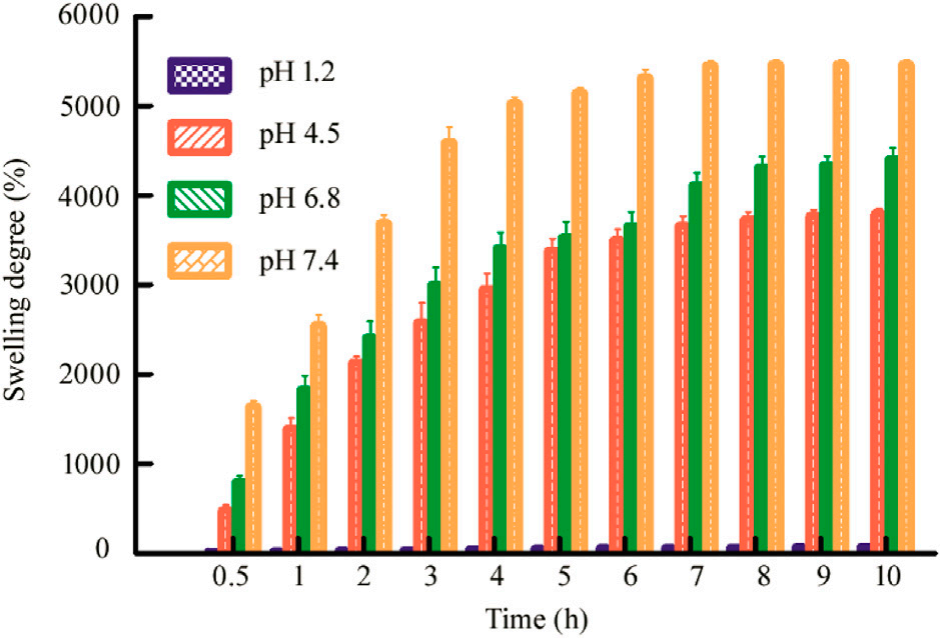
Swelling degree of dry Alg/CS/Car microbeads beads in pH 1.2, pH 4.5, pH 6.8, and pH 7.4. Data are mean ± SD (*n* = 3) of three independent experiments.

The swelling behavior is a very important property of the drug delivery carrier because it has a great influence on the controlled release behavior of the drug, so this section conducts swelling experiments on the hydrogel microspheres (Sun et al., 2019). As shown in Figure 5, it can be seen that the swelling degree of the hydrogel beads at different pH basically maintains a trend similar to the release rate, and also has significant pH dependence. Under the condition of pH 1.2 of the medium, the shape and size of the hydrogel beads remained basically unchanged. Under pH 4.5 and pH 6.8, as time goes by, the hydrogel beads gradually became larger, and the swelling degree of the beads under the condition of pH 6.8 at the corresponding time point was always greater than that of the beads under the pH 4.5. Under the pH 7.4 medium, it had a maximum swelling degree at each time point. Therefore, it can be seen that under different pH, different swelling degrees of hydrogel beads can achieve different drug release rates.

### Cytotoxic Study

Cytotoxicity is the most important factor in assessing the eligibility of drug delivery vehicles. When treating colitis, cytotoxicity is a consideration (Liu et al., 2020). Here, we used Raw 264.7 macrophages and HT-29 cells to evaluate the CCK-8 *in vitro* cytotoxicity experiments on the hydrogel beads and MSZ inclusion compounds at different concentrations (Pan et al., 2019). Raw 264.7 macrophages were chosen because it plays a vital role in maintaining the body’s intestinal mucosal surface microbial homeostasis and continuous intestinal epithelial cell renewal. It is not only an important part of innate immunity, but also involved in the pathological process of inflammatory bowel disease (IBD). Use HT-29 cells to further prove their biocompatibility.

**FIGURE 6:**
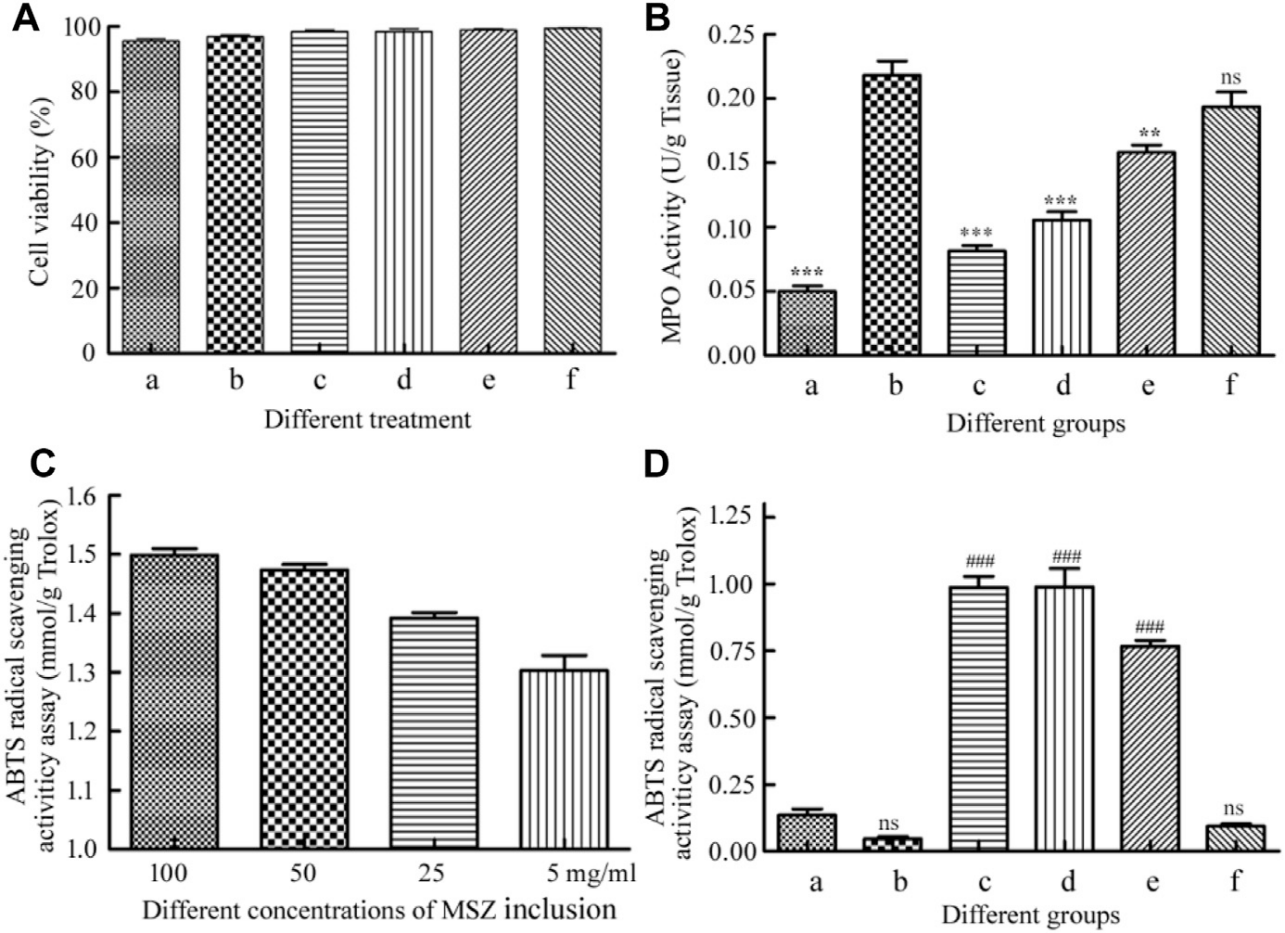
**(A)** The cytotoxicity of the HP-β-CD/MSZ inclusion complex, the blank hydrogel, and the drug-loaded hydrogel by the CCK-8 assay. **(B)** Colonic MPO activities. **(C)** ABTS radical cation scavenging activity of the HP-β-CD/MSZ inclusion complex with different concentrations. **(D)** ABTS radical cation scavenging activity of mice serum expressed as trolox equivalents. In **(B**,**D)**, a, normal; b, DSS; c, the positive drug group; d, the MSZ-loaded hydrogel group, e, the HP-β-CD/MSZ inclusion complex group; and f, the blank hydrogel group. The data in **(A**,**C)** are the mean ± SD of three independent experiments (*n* = 3). The data in **(B**,**D)** are the mean ± SD of six data (*n* = 6); compare to the DSS group, **p < 0.01, ***p < 0 .001; compared to normal group, ^###^ *p* < 0.001; ns, no significance.

The concentrations of the HP-β-CD/MSZ inclusion complex were selected as 200, 100, 50, and 25 mg/ml, which were represented by a, b, c and d, respectively, in Figure 6A. The hydrogel beads were crushed and treated with suspension, and e and f represented a blank hydrogel and a drug-loaded hydrogel, respectively. Finally, the cell survival rate was recorded using the following formula: cell survival rate = [(As−Ab)/(Ac−Ab)] × 100%; As, Ab, and Ac are the absorbance values of the experimental, blank, and control wells, respectively. A CCK-8 analysis showed that the hydrogel had basically no effect on the survival rate of Raw 264.7 macrophages. The MSZ inclusion compound had no effect on the Raw 264.7 macrophages at low concentrations, but slightly harmed the cells at the highest concentration, but the cell survival rate was also above 95%. They also have relatively little toxicity to HT-29 cells (Supplementary Figure S3). The results confirmed that the two showed low cytotoxicity to the cell line studied, and it was concluded that the drug-loaded hydrogel had good biocompatibility, and the microspheres were safe for drug delivery applications.

### Myeloperoxidase Activity

MPO, mainly produced by neutrophils, is usually related to the presence of neutrophils in the mucosa and submucosa of inflammatory tissues, and is considered an important indicator of inflammation in ulcerative colitis (Javanbakht et al., 2020). As shown in Figure 6B, compared with the healthy control group, the MPO activity of the colitis group was significantly increased (*p*<0.001). The MPO activity of the blank hydrogel treatment group and the MSZ inclusion compound treatment group was slightly lower than that of the DSS group; however, compared with the DDS group, the positive control group and the hydrogel drug-loaded group significantly inhibited MPO activity (*p*<0.001), indicating that MSZ can be effectively delivered to the inflamed colon and has excellent therapeutic activity.

### *In vitro* Analysis of Antioxidant Activity

It has been reported that MSZ has the characteristics of an effective oxygen free radical scavenger, so it has a strong antioxidant capacity and is expected to be used as an antioxidant (Joshi et al., 2005; Managlia et al., 2013). In order to evaluate the antioxidant activity of MSZ, ABTS free radical cation scavenger was used. As shown in Figure 6C, among all the tested compounds, the MSZ inclusion compound at the highest concentration showed the highest ABTS free radical cation scavenging activity, and the inhibition rate obtained by the measurement was the same as that of the 1.5 mM Trolox. With the gradual decrease of MSZ concentration, the inhibition rate also slightly decreased. As shown in Figure 6D, from the results of the eyeball blood serum measurement of the mice in the experimental group, it can be seen that the hydrogel-loaded MSZ group showed greater antioxidant capacity than the other two groups, it indicated that the MSZ contained in the MSZ hydrogel had a good release and played a better antioxidant capacity.

### Histopathologic Analysis

**FIGURE 7:**
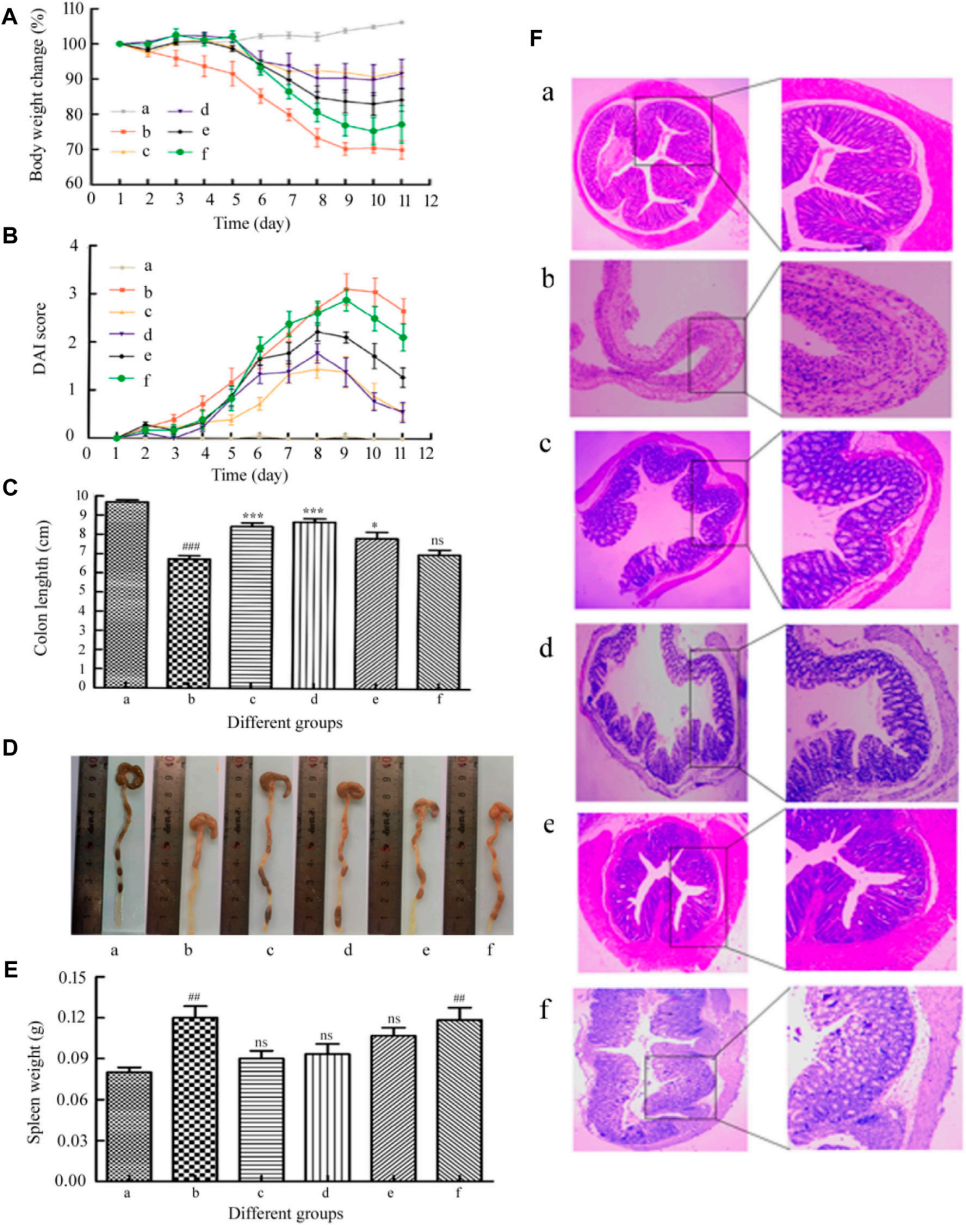
**(A)** Loss of basal body weight under different treatments during the disease process. **(B)** DAI under different treatments. **(C**,**D)** Changes of colon length in each group of mice. **(E)** Spleen weight of mice in each group. **(F)** Representative H&E staining of the mouse colon. a, normal; b, DSS; c, the positive drug group; d, the MSZ-loaded hydrogel group, e, the HP-β-CD/MSZ inclusion complex group; and f, the blank hydrogel group. Data are the mean ± SD of six data (*n* = 6). Compare to the DSS group, *p < 0 0.05, **p < 0.01, ***p < 0 .001; compared to normal group, #*p* < 0.05, ##*p* < 0.01, ###*p* < 0.001. **(C)** ns, no significance, compare to the DSS group; **(E)** ns, no significance, compare to normal group.

In order to study the therapeutic effect of acute colitis, we established a mouse model of acute colitis and further treated the mice. A clinical score that assesses weight loss, stool consistency, and anal bleeding was used to quantify colitis activity (DAI assessment). The body weight changes of mice in each group are shown in Figure 7A. Before DSS administration, there was no difference in body weight among the groups. The weight of the healthy control group increased slightly. Compared with this, 2.5% DSS in drinking water can significantly reduce weight, leading to colitis. Compared with the colitis control group, the MSZ hydrogel treatment group had a significant improvement in body weight. Although the blank hydrogel and the HP-β-CD/MSZ group had better body weight than the colitis group, they were not as good as the hydrogel drug group. The severity of colitis was scored by DAI. As shown in Figure 7B, compared with normal mice, all colitis mice recorded different levels of DAI. The DAI score of model mice was the highest, the DAI of mice treated with an MSZ inclusion compound and a blank hydrogel decreased slightly, while the reduction of DAI of mice treated with the MSZ hydrogel and commercial MSZ was the most obvious, slightly higher than that of the normal group.

The length of the colon is an important parameter to evaluate the anti-inflammatory activity in the colitis model; the spleen is an important organ of the body’s immune system, and the increase in spleen weight is also used as a sign of the severity of inflammation (Mladenovska et al., 2007). After the last drug treatment, all mice were anesthetized to death. Surgery was conducted to open the abdominal cavity, remove the colon (with cecum) and spleen (X. Zhou et al., 2020b). As shown in Figures 7C,D, the average colorectal length of the mice in the normal control group was close to 10.0 cm, the colon length of the mice in the DSS control group is significantly shorter than that of the mice in the healthy control group (*p* < 0.001). This is the main indicator of colitis induced by direct administration. Therefore, the expected therapeutic index of the therapeutic effect will be that the drug-treated colon basically returns to the initial value. Compared with the DSS group, the hydrogel drug-loaded group and the positive drug group significantly reduced the shortening of the colon length (*p* < 0.001). Although the mice in all experimental treatment groups had a shorter colon length compared with the mice in the healthy control group, the mice in the hydrogel treatment group showed the colon length was longer than that of the mice in the other experimental treatment groups. For example, the average the length of the colon and the rectum of mice in the MSZ direct administration group and the blank hydrogel group are 7.9 and 7.2 cm, respectively. Regarding the weight of the spleen, the weight of the spleen of the mice in the colitis control group was significantly higher than that of the healthy control group (*p* < 0.01). The weight of the spleen of the mice treated with HP-β-CD/MSZ and blank hydrogel was slightly reduced. However, the average spleen weight of the mice treated with MSZ loaded with hydrogel was lower than that of the other two groups, indicating that MSZ loaded with hydrogel improved colitis to a greater extent.

### Microscopic Assessment of Colitis

In order to further verify the treatment effect, H&E staining was used for histological evaluation of colon tissues in all groups to observe the changes in the anatomical structure of the colon wall (Fang et al., 2018). As expected, as shown in Figure 7F, the colon tissues of the healthy control mice showed healthy histological features and showed no signs of destructive morphology. On the contrary, compared with normal tissues, the tissue sections of the colitis DSS control group showed obvious destruction of the epithelial barrier and the accumulation of immune cells were obviously destroyed, crypt deformation, loss of goblet cells, inflammatory cell infiltration, and severe mucosal damage. Compared with mice in the DSS control group, the inflammatory manifestations of mice in all experimental treatment groups were relatively reduced. The DSS-induced a series of symptoms, such as injury and inflammatory cell infiltration, were significantly reversed after administration of commercially available MSZ and treatment with the MSZ hydrogel, showing a morphological structure similar to that of healthy mouse tissue, which indicates epithelial repair and inflammation alleviation. These results indicate that MSZ ameliorated DSS-induced colitis in a mouse colitis model.

### *In vivo* Imaging Analysis

**FIGURE 8:**
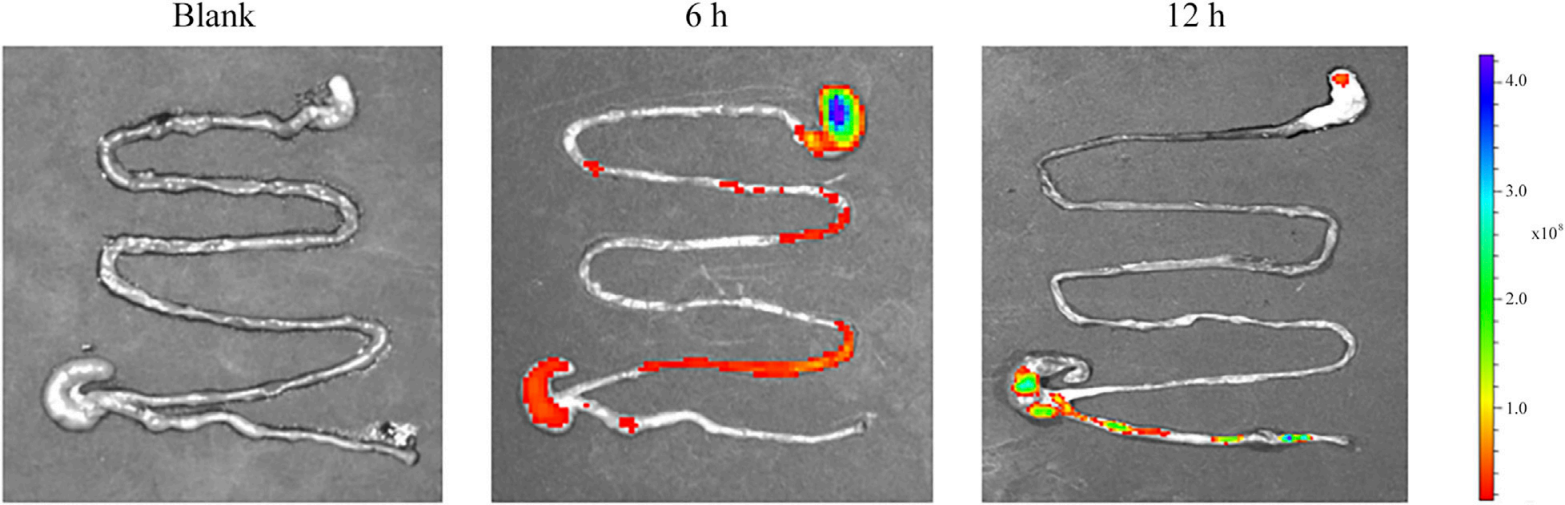
Representative *ex vivo* images of the GIT after oral administration of blank and RBITC-loaded hydrogel, showing colon-targeted drug delivery and accumulation potential.

We assumed that after oral administration, the MSZ hydrogel in the colitis model swells and aggregates more in the inflamed colon. In order to test this hypothesis, in this study, we used a small animal *in vivo* 3D imaging system to perform *ex vivo* fluorescence imaging to evaluate the biodistribution of the hydrogel in the animal, in order to explore the ability of the hydrogel to target the colon. By covalently binding rhodamine B isothiocyanate (RBITC) to a hydrogel containing chitosan, the small animal *in vivo* imager can perform real-time and quantitative analysis of RBITC dye-labeled therapeutics. After 6 and 12 h of administration, the GIT was removed for imaging analysis. As shown in Figure 8, blank hydrogel mice were used to identify the role of carrier materials, and no fluorescent signal was observed. In contrast, RBITC-loaded hydrogels produced fluorescent signals at both time points. At 6 h, there was a relatively strong fluorescence signal at the upper part of the colon of colitis mice, indicating that the hydrogel had not yet reached the colon through the *in vivo* process. In colitis mice, there is a strong fluorescence signal at 12 h, indicating that the hydrogel has colon-targeting and strong retention ability for colitis mice.

## Conclusion

In this study, the HP-β-CD/MSZ inclusion compound was wrapped with layers of hydrogels formed by Alg, Cs, and Car. After drying, the beads could reach about one size for oral gavage, and Alg/Cs/kC microcapsules can effectively encapsulate HP-β-CD/MSZ. The swelling behavior *in vivo* showed that the prepared beads depended on the pH of the composite hydrogel microbeads, that is, they had different swelling capabilities in different pH environments, so the drug release behavior will change significantly. Compared with the positive drug group, the MSZ hydrogel showed similar therapeutic effect, and the Alg/Cs/kC composite microsphere material has excellent biodegradability, which is beneficial to its practical application in human body. Overall, the obtained results indicated that the hydrogel-embedded MSZ complex can be used as a promising oral therapeutic agent for the treatment of acute colitis.

## Data Availability

The original contributions presented in the study are included in the article/Supplementary Material; further inquiries can be directed to the corresponding authors.
