# Supplementary material for: Preparation of MSZ Hydrogel and Its Treatment of Colitis
Source: Front Pharmacol. 2021 Oct 8;12:706401. doi: 10.3389/fphar.2021.706401 (PMC8531416; doi:10.3389/fphar.2021.706401)
Supplement: Supplementary file 1 [file DataSheet1.PDF]

Supplementary Materials

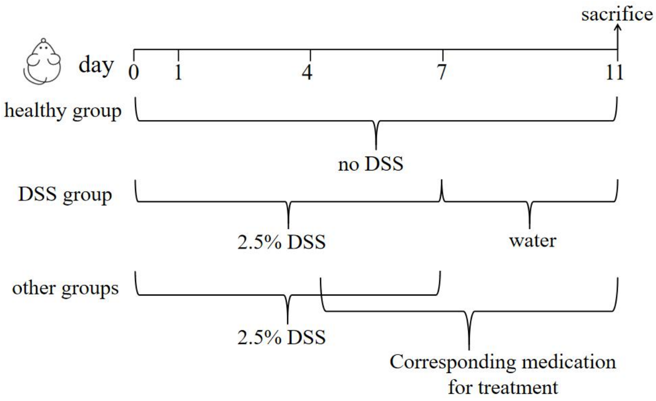

Figure S1. Flow chart.

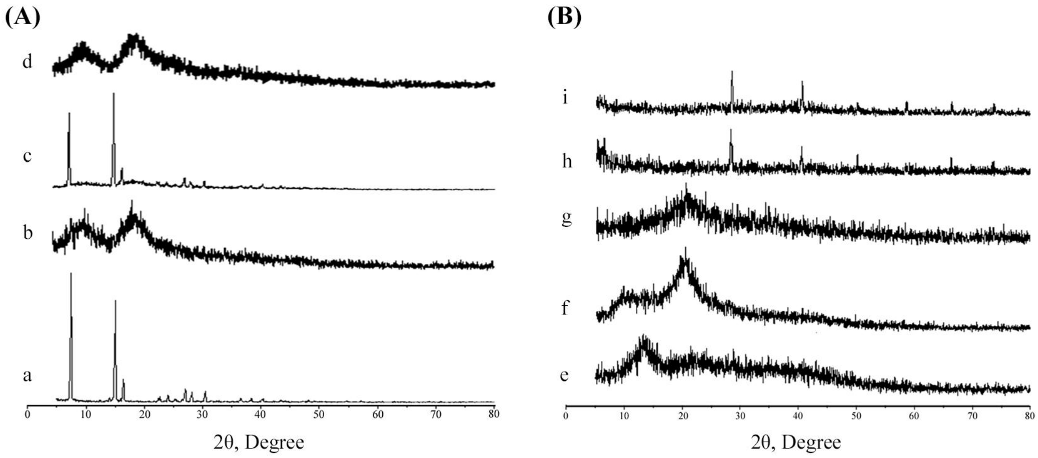

Figure S2. XRD spectra. a, MSZ; b, HP-β-CD; c, physical mixture of MSZ and HP-β-CD; d, inclusion complex of MSZ and HP-β-CD; e, Alg; f, Cs; g, κ-Car; h, blank hydrogel; and i, drug loaded hydrogel.

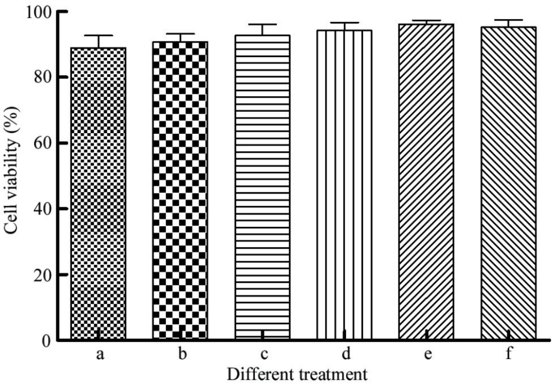

Figure S3. Cytotoxicity test of HT-29 cells. Data are mean ± SD (n = 3) of three independent experiments.
